# Hafnium germanium telluride

**DOI:** 10.1107/S1600536808011380

**Published:** 2008-04-30

**Authors:** Gyung-Joo Jang, Hoseop Yun

**Affiliations:** a Division of Energy Systems Research and Department of Chemistry, Ajou University, Suwon 443-749, Republic of Korea

## Abstract

The title hafnium germanium telluride, HfGeTe_4_, has been synthesized by the use of a halide flux and structurally characterized by X-ray diffraction. HfGeTe_4_ is isostructural with stoichiometric ZrGeTe_4_ and the Hf site in this compound is also fully occupied. The crystal structure of HfGeTe_4_ adopts a two-dimensional layered structure, each layer being composed of two unique one-dimensional chains of face-sharing Hf-centered bicapped trigonal prisms and corner-sharing Ge-centered tetra­hedra. These layers stack on top of each other to complete the three-dimensional structure with undulating van der Waals gaps.

## Related literature

For the synthesis, crystal structure, and electronic structure of Hf_0.85_GeTe_4_, see: Mar & Ibers (1993 ▶). For the synthesis and structure of ZrGeTe_4_, see: Lee *et al.* (2007 ▶). The title compound, HfGeTe_4_, is isostructural with Hf_0.85_GeTe_4_ and ZrGeTe_4_. However the Hf site in HfGeTe_4_ is fully occupied. For related literature, see: Furuseth *et al.* (1973 ▶); Gelato & Parthé (1987 ▶); Smith & Bailey (1957 ▶); Zhao & Parthé (1990 ▶).

## Experimental

### 

#### Crystal data

HfGeTe_4_
*M* *_r_* = 761.48
Orthorhombic, 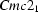

*a* = 3.97951 (17) Å
*b* = 15.9530 (7) Å
*c* = 10.9731 (7) Å
*V* = 696.63 (6) Å^3^
*Z* = 4
Mo *K*α radiation
μ = 35.50 mm^−1^
*T* = 290 (1) K
0.30 × 0.02 × 0.02 mm

#### Data collection

Rigaku R-AXIS RAPID diffractometer
Absorption correction: numerical (*NUMABS*; Higashi, 2000 ▶) *T*_min_ = 0.425, *T*_max_ = 0.510
3348 measured reflections
910 independent reflections
878 reflections with *I* > 2σ(*I*)
*R*_int_ = 0.060

#### Refinement

*R*[*F* ^2^ > 2σ(*F* ^2^)] = 0.023
*wR*(*F* ^2^) = 0.054
*S* = 0.97
910 reflections
38 parameters
1 restraint
Δρ_max_ = 1.55 e Å^−3^
Δρ_min_ = −2.00 e Å^−3^
Absolute structure: Flack (1983 ▶), 431 Friedel pairs
Flack parameter: 0.008 (14)

### 

Data collection: *RAPID-AUTO* (Rigaku, 2006 ▶); cell refinement: *RAPID-AUTO*; data reduction: *RAPID-AUTO*; program(s) used to solve structure: *SHELXS97* (Sheldrick, 2008 ▶); program(s) used to refine structure: *SHELXL97* (Sheldrick, 2008 ▶); molecular graphics: locally modified version of *ORTEP* (Johnson, 1965 ▶); software used to prepare material for publication: *WinGX* (Farrugia, 1999 ▶).

## Supplementary Material

Crystal structure: contains datablocks global, I. DOI: 10.1107/S1600536808011380/gw2040sup1.cif

Structure factors: contains datablocks I. DOI: 10.1107/S1600536808011380/gw2040Isup2.hkl

Additional supplementary materials:  crystallographic information; 3D view; checkCIF report

## Figures and Tables

**Table:** 

Hf—Ge^i^	2.8286 (15)
Hf—Te3^ii^	2.9454 (8)
Hf—Te3^iii^	2.9454 (8)
Hf—Te1^iii^	2.9524 (7)
Hf—Te1^ii^	2.9524 (7)
Hf—Te2^iii^	2.9825 (8)
Hf—Te2^ii^	2.9825 (8)
Hf—Te4^iv^	3.0312 (11)
Ge—Te4^v^	2.6761 (10)
Ge—Te4^vi^	2.6761 (10)
Ge—Te3	2.6955 (17)
Te1—Te2	2.7387 (13)

**Table:** 

Te4^v^—Ge—Te4^vi^	96.07 (5)
Te4^v^—Ge—Te3	92.35 (4)
Te4^v^—Ge—Hf^iv^	123.85 (4)
Te3—Ge—Hf^iv^	120.07 (5)

Symmetry codes: (i) 
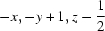
; (ii) 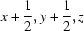; (iii) 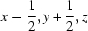; (iv) 
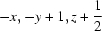
; (v) 
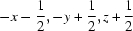
; (vi) 
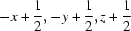
.

**Fig. 1.**
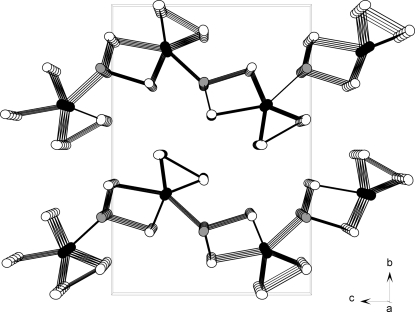
View of HfGeTe4 down the a axis, showing the layered nature of the compound. Filled, grey, and open circles represent Hf, Ge, and Te atoms, respectively. Displacement ellipsoids are drawn at the 90% probability level.

**Fig. 2.**
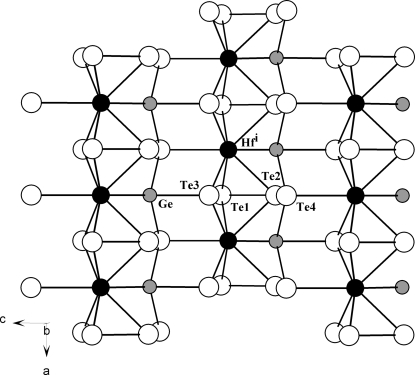
View of HfGeTe4 down the b axis, showing a two-dimensional layer. Atoms are as marked in Fig. 1. [Symmetry code: (i) -1/2 + x, -1/2 + y, z]

## supplementary crystallographic information

### Comment

A view of the structure of HfGeTe_4_ down the *a* axis in Fig. 1 shows the layered nature of the compound. Fig 2. shows that an individual layer is composed of two unique one-dimensional chains of face-sharing Hf-centered bicapped trigonal prisms and corner-sharing Ge-centered tetrahedra. The title compound is isostructural with Hf_0.85_GeTe_4_ (Mar & Ibers, 1993) and ZrGeTe_4 _(Lee *et al.*, 2007) and the detailed descriptions of this structural type have been given previously.

The Hf atom is surrounded by six Te atoms in a trigonal prismatic manner. The vertices of two base sides of the prism are composed of six Te atoms. Atoms Te1, Te2, and Te3 form a triangle that is isosceles and the short Te1—Te2 distance (2.739 (1) Å) is typical of (Te—Te)^2-^ pair (Furuseth *et al.*, 1973). An additional Te4 and Ge atoms cap two of the rectangular faces of the trigonal prism to complete the bicapped trigonal prismatic coordination. These trigonal prisms share their triangular faces to form an infinite chain, _∞_^1^[HfGeTe_4_] along the *a* axis. The Ge atom is surrounded by three Te and one Hf atoms in distorted tetrahedral fashion. These tetrahedra share their corners through the Te4 atom to form an infinite chain.

These bicapped trigonal prismatic and the tetrahedral chains are fused through Hf—Ge bonds to form a double chain and finally these chains are linked along the *c* axis to complete the two-dimensional layer. These layers then stack on top of each other to form the three-dimensional structure with undulating van der Waals gaps shown in Fig. 1.

The Hf—Ge bond distance is 2.829 (2) Å, which is comparable with those found in other hafnium germanides (HfGe2, 2.78–2.87 Å (Smith & Bailey, 1957); Hf_5_Ge_4_, 2.82 Å (Zhao & Parthé, 1990)).

### Experimental

HfGeTe_4_ was prepared from a reaction of Hf(CERAC 99.8%), Ge(CERAC 99.999%), and Te(CERAC 99.95%) in an elemental ratio of 1:1:4 in the presence of KCl(Aldrich 99.99%) as flux. The mass ratio of reactants and flux was 1:3. The starting materials were placed in a fused-silica tube. The tube was evacuated to 10 ^-3^ Torr, sealed, and heated to 973 K at a rate of 15 K/hr, where it was kept for 72 hrs. The tube was cooled at a rate of 10 K/hr to 373 K and the furnace was shut off. Air- and water-stable metallic shiny needle-shaped crystals were isolated after the flux was removed with water. Qualitative analysis of the crystals with an EDAX-equipped scanning electron microscope indicated the presence of Hf, Ge, and Te. No other element was detected.

### Refinement

Refinement with the positional parameters taken from the ZrGeTe_4_ structure (Lee *et al.*, 2007) gave the value of the Flack parameter (Flack, 1983) of *x*=0.96 (4) (wR2=0.128), which suggests that the absolute structure should be incorrect. Refinement of the inverse structure which is in agreement with the selected setting of this work leads to *x*=-0.02 (6) and significantly better reliability factor (wR2=0.054). The structure was standardized by means of the program *STRUCTURE TIDY* (Gelato & Parthé, 1987). The nonstoichiometry of the Hf site in the title compound was checked by refining the occupancy and anisotropic displacement parameters of Hf while those of the other atoms were fixed. With the nonstoichiometric model, both parameter were not changed significantly and the residuals (wR2, R1 indices) remained the same. The highest peak(1.55 e/Å^-3^) and the deepest hole (-2.00 e/ Å^-3^) are 0.98 Å and 0.78 Å from the atom Hf, respectively.

### Crystal data

**Table:**

HfGeTe_4_	*F*(000) = 1248
*M**_r_* = 761.48	*D*_x_ = 7.26 Mg m^−^^3^
Orthorhombic, *C**m**c*2_1_	Mo *K*α radiation, λ = 0.71073 Å
Hall symbol: C 2c -2	Cell parameters from 3229 reflections
*a* = 3.97951 (17) Å	θ = 3.2–27.5°
*b* = 15.9530 (7) Å	µ = 35.50 mm^−^^1^
*c* = 10.9731 (7) Å	*T* = 290 K
*V* = 696.63 (6) Å^3^	Needle, metallic silver
*Z* = 4	0.30 × 0.02 × 0.02 mm

### Data collection

**Table:**

Rigaku R-AXIS RAPID diffractometer	878 reflections with *I* > 2σ(*I*)
ω scans	*R*_int_ = 0.060
Absorption correction: numerical (*NUMABS*; Higashi, 2000)	θ_max_ = 27.5°, θ_min_ = 3.2°
*T*_min_ = 0.425, *T*_max_ = 0.510	*h* = −4→5
3348 measured reflections	*k* = −20→19
910 independent reflections	*l* = −14→14

### Refinement

**Table:**

Refinement on *F*^2^	*w* = 1/[σ^2^(*F*_o_^2^) + (0.0001*P*)^2^] where *P* = (*F*_o_^2^ + 2*F*_c_^2^)/3
Least-squares matrix: full	(Δ/σ)_max_ = 0.001
*R*[*F*^2^ > 2σ(*F*^2^)] = 0.023	Δρ_max_ = 1.55 e Å^−^^3^
*wR*(*F*^2^) = 0.054	Δρ_min_ = −2.00 e Å^−^^3^
*S* = 0.97	Extinction correction: *SHELXL97* (Sheldrick, 2008), Fc^*^=kFc[1+0.001xFc^2^λ^3^/sin(2θ)]^-1/4^
910 reflections	Extinction coefficient: 0.00231 (14)
38 parameters	Absolute structure: Flack (1983), 431 Friedel pairs
1 restraint	Flack parameter: 0.008 (14)

### Special details

**Table:**

Geometry. All e.s.d.'s are estimated using the full covariance matrix. The cell e.s.d.'s are taken into account individually in the estimation of e.s.d.'s in distances, angles and torsion angles; correlations between e.s.d.'s in cell parameters are only used when they are defined by crystal symmetry.

### Fractional atomic coordinates and isotropic or equivalent isotropic displacement parameters (Å^2^)

**Table:**

	*x*	*y*	*z*	*U*_iso_*/*U*_eq_	
Hf	0.0000	0.65202 (3)	0.22820 (5)	0.01067 (17)	
Ge	0.0000	0.22772 (8)	0.53878 (13)	0.0113 (3)	
Te1	0.0000	0.01673 (5)	0.25689 (9)	0.0129 (2)	
Te2	0.0000	0.10126 (5)	0.03966 (9)	0.0126 (2)	
Te3	0.0000	0.27773 (5)	0.30414 (8)	0.0102 (2)	
Te4	0.0000	0.38127 (5)	0.00017 (8)	0.0106 (2)	

### Atomic displacement parameters (Å^2^)

**Table:**

	*U*^11^	*U*^22^	*U*^33^	*U*^12^	*U*^13^	*U*^23^
Hf	0.0113 (3)	0.0107 (3)	0.0100 (3)	0	0	0.0000 (2)
Ge	0.0118 (6)	0.0112 (7)	0.0108 (8)	0	0	0.0012 (6)
Te1	0.0140 (4)	0.0100 (4)	0.0147 (5)	0	0	0.0008 (4)
Te2	0.0142 (4)	0.0130 (4)	0.0106 (5)	0	0	−0.0012 (4)
Te3	0.0116 (3)	0.0093 (4)	0.0097 (4)	0	0	−0.0010 (3)
Te4	0.0118 (4)	0.0087 (4)	0.0111 (5)	0	0	0.0002 (3)

### Geometric parameters (Å, °)

**Table:**

Hf—Ge^i^	2.8286 (15)	Ge—Hf^iv^	2.8286 (15)
Hf—Te3^ii^	2.9454 (8)	Te1—Te2	2.7387 (13)
Hf—Te3^iii^	2.9454 (8)	Te1—Hf^vii^	2.9524 (7)
Hf—Te1^iii^	2.9524 (7)	Te1—Hf^viii^	2.9524 (7)
Hf—Te1^ii^	2.9524 (7)	Te2—Hf^vii^	2.9825 (8)
Hf—Te2^iii^	2.9825 (8)	Te2—Hf^viii^	2.9825 (8)
Hf—Te2^ii^	2.9825 (8)	Te3—Hf^viii^	2.9454 (8)
Hf—Te4^iv^	3.0312 (11)	Te3—Hf^vii^	2.9454 (8)
Ge—Te4^v^	2.6761 (10)	Te4—Ge^ix^	2.6761 (10)
Ge—Te4^vi^	2.6761 (10)	Te4—Ge^x^	2.6761 (10)
Ge—Te3	2.6955 (17)	Te4—Hf^i^	3.0312 (11)
			
Ge^i^—Hf—Te3^ii^	75.29 (3)	Te3^iii^—Hf—Te4^iv^	80.84 (2)
Ge^i^—Hf—Te3^iii^	75.29 (3)	Te1^iii^—Hf—Te4^iv^	76.52 (3)
Te3^ii^—Hf—Te3^iii^	84.99 (3)	Te1^ii^—Hf—Te4^iv^	76.52 (3)
Ge^i^—Hf—Te1^iii^	125.04 (3)	Te2^iii^—Hf—Te4^iv^	129.45 (2)
Te3^ii^—Hf—Te1^iii^	157.35 (3)	Te2^ii^—Hf—Te4^iv^	129.45 (2)
Te3^iii^—Hf—Te1^iii^	90.704 (18)	Te4^v^—Ge—Te4^vi^	96.07 (5)
Ge^i^—Hf—Te1^ii^	125.04 (3)	Te4^v^—Ge—Te3	92.35 (4)
Te3^ii^—Hf—Te1^ii^	90.704 (18)	Te4^vi^—Ge—Te3	92.35 (4)
Te3^iii^—Hf—Te1^ii^	157.35 (3)	Te4^v^—Ge—Hf^iv^	123.85 (4)
Te1^iii^—Hf—Te1^ii^	84.75 (3)	Te4^vi^—Ge—Hf^iv^	123.85 (4)
Ge^i^—Hf—Te2^iii^	71.00 (3)	Te3—Ge—Hf^iv^	120.07 (5)
Te3^ii^—Hf—Te2^iii^	146.29 (3)	Te2—Te1—Hf^vii^	63.08 (2)
Te3^iii^—Hf—Te2^iii^	86.01 (2)	Te2—Te1—Hf^viii^	63.08 (2)
Te1^iii^—Hf—Te2^iii^	54.96 (3)	Hf^vii^—Te1—Hf^viii^	84.75 (3)
Te1^ii^—Hf—Te2^iii^	108.97 (3)	Te1—Te2—Hf^vii^	61.96 (2)
Ge^i^—Hf—Te2^ii^	71.00 (3)	Te1—Te2—Hf^viii^	61.96 (2)
Te3^ii^—Hf—Te2^ii^	86.01 (2)	Hf^vii^—Te2—Hf^viii^	83.69 (3)
Te3^iii^—Hf—Te2^ii^	146.29 (3)	Ge—Te3—Hf^viii^	93.94 (3)
Te1^iii^—Hf—Te2^ii^	108.97 (3)	Ge—Te3—Hf^vii^	93.94 (3)
Te1^ii^—Hf—Te2^ii^	54.96 (3)	Hf^viii^—Te3—Hf^vii^	84.99 (3)
Te2^iii^—Hf—Te2^ii^	83.69 (3)	Ge^ix^—Te4—Ge^x^	96.07 (5)
Ge^i^—Hf—Te4^iv^	147.38 (4)	Ge^ix^—Te4—Hf^i^	92.41 (4)
Te3^ii^—Hf—Te4^iv^	80.84 (2)	Ge^x^—Te4—Hf^i^	92.41 (4)

Symmetry codes: (i) −*x*, −*y*+1, *z*−1/2; (ii) *x*+1/2, *y*+1/2, *z*; (iii) *x*−1/2, *y*+1/2, *z*; (iv) −*x*, −*y*+1, *z*+1/2; (v) −*x*−1/2, −*y*+1/2, *z*+1/2; (vi) −*x*+1/2, −*y*+1/2, *z*+1/2; (vii) *x*+1/2, *y*−1/2, *z*; (viii) *x*−1/2, *y*−1/2, *z*; (ix) −*x*−1/2, −*y*+1/2, *z*−1/2; (x) −*x*+1/2, −*y*+1/2, *z*−1/2.
